# Characterization of Chitosan Films Modified Using Caffeic Acid and a Neutralization Process

**DOI:** 10.3390/ma16145038

**Published:** 2023-07-17

**Authors:** Marta Szulc, Katarzyna Lewandowska

**Affiliations:** Department of Biomaterials and Cosmetic Chemistry, Faculty of Chemistry, Nicolaus Copernicus University in Toruń, Gagarin 7, 87-100 Toruń, Poland

**Keywords:** chitosan, cross-linking, surface properties, alkali treatment

## Abstract

In the context of the growing interest in biopolymer-based materials for various applications, this study aimed to enhance the properties of chitosan (CS, a derivative of chitin) films by incorporating caffeic acid, a polyphenol compound known for its multiple health benefits. The objective was to improve the mechanical parameters of the resulting films, including surface roughness and elasticity. CS was combined with caffeic acid and then underwent a neutralization process. The modified films exhibited potential for use in soft tissue engineering, where increased elasticity and surface roughness are desirable characteristics. The main methods employed to evaluate the structure and properties of the films included mechanical analysis, infrared spectroscopy, scanning electron microscopy, atomic force microscopy, thermogravimetric analysis, contact angle measurement, and swelling behavior. The study’s main findings revealed significant alterations in the mechanical properties and surface morphology of the films. The main conclusions drawn from the study suggest that interactions between caffeic acid and CS hold promise for the development of advanced biomaterials in medicine, tissue engineering, and cosmetic formulations. However, a deeper understanding of these interactions is necessary to optimize the material properties and unlock their full potential.

## 1. Introduction

Over the past decade, there has been a significant surge in the utilization of biopolymer-based materials. Among the most notable and widely researched biopolymers is chitosan (CS), which is derived from chitin. CS can be obtained from the shells of marine animals, including crabs, shrimp, and squid [1,2]. This natural polymer possesses a wide range of desirable properties, including biodegradability, biocompatibility, and antibacterial activity. It is also soluble in acids, particularly organic acids such as acetic acid or lactic acid. However, the properties of CS-based materials are influenced by several factors, including the molecular weight of CS, the degree of deacetylation, and the choice of solvent [3,4,5,6,7]. Despite these advantageous characteristics, CS does have a limitation in terms of its relatively low mechanical strength [8].

To overcome this drawback and enhance the performance of CS materials, numerous modification strategies have been investigated. These approaches often involve the incorporation of additional polymers as well as low molecular weight compounds and the utilization of cross-linking techniques [9,10,11,12,13]. Cross-linking agents, such as citric acid, glutaric dialdehyde, genipin, and phenolic compounds, are widely utilized to improve the mechanical properties, water-sensitivity, barrier properties, oxygen/water vapor transmission rates, and antimicrobial properties of polymer films [14,15,16]. Cross-linking is a process that involves connecting polymer chains through covalent or non-covalent bonds, resulting in the formation of a three-dimensional network. This network reduces the mobility of the polymer chains and enhances their mechanical and barrier properties, as well as their water resistance. One intriguing compound that has attracted attention due to its potential synergistic effects with CS is caffeic acid (CA), a polyphenol compound. CA is well known for its antibacterial, antiviral, antioxidant, and anti-inflammatory properties, while also demonstrating promising anti-cancer effects in several studies [17,18,19].

Various studies have investigated materials derived from the combination of CS and CA. For example, Haijie Xu et al. prepared CS films by incorporating CA and Fe^3+^. The resulting films exhibited enhanced mechanical properties, including a higher Young’s modulus, as well as improved antibacterial and antioxidant properties [20]. Claudia Nunes et al. developed CS/CA/genipin films through a neutralization process, which resulted in notable improvements in antioxidant properties and reduced acid solubility [21]. Furthermore, Beata Kaczmarek-Szczepańska et al. successfully obtained CS/CA films modified with poly(ethylene glycol), demonstrating favorable antioxidant and antibacterial properties [22].

To further enhance the characteristics of CS films, a commonly employed technique is the use of a neutralization process. This method typically involves neutralizing the films using a sodium hydroxide (NaOH) solution [23,24]. The neutralization step significantly impacts the mechanical properties of the films and helps reduce the swelling factor. Alternatively, buffers that correspond to the acids in which CS has been dissolved can also be utilized [24,25]. It is worth noting that there is no existing literature describing a combination of both approaches, namely the addition of CA and subsequent neutralization of the film.

In this study, our objective was to conduct a comprehensive evaluation of the structure and properties of CA films using a range of analytical techniques. These techniques include mechanical analysis, infrared spectroscopy, scanning electron microscopy, atomic force microscopy, thermogravimetric analysis, contact angle measurement, and swelling behavior. By performing these assessments both before and after the neutralization process, we aimed to gain a deeper understanding of the effects and potential synergies between the addition of CA and the neutralization step. The insights obtained from this research represented a significant advancement in the investigation of cross-linked CS materials. Ultimately, this knowledge will contribute to the design and development of innovative biomaterials for diverse applications, including medicine, tissue engineering, and cosmetics.

## 2. Materials and Methods

### 2.1. Materials

The CS powder used in this study was obtained from the Marine Fisheries Research Institute (Gdynia, Poland). It had a degree of deacetylation of 80% (estimated through potentiometric titration) and a viscosity average molecular weight of 1382 kg/mol, which was determined using the Mark–Houwink–Sakurada equation. CA was purchased from Pol-aura (Morąg, Poland). All the other reagents utilized in this study were of analytical grade and were obtained from POCh (Avantor, Gliwice, Poland) and Chempur (Piekary Śląskie, Poland). These chemicals were used as received without requiring any further treatment.

### 2.2. Film Preparation and Neutralization Process

CS was dissolved in a 0.5 mol/L acetic acid at a concentration of 2% *w*/*v*. CA powder was added to the polymer solutions at 20% *w*/*w* relative to the polymer mass and magnetically stirred until fully dissolved (24 h at room temperature). The resulting solutions were poured into square Petri dishes (100 × 15 mm) and allowed to dry at room temperature under conditions of 40–50% humidity for a period of 72 h.

**Figure 1:**
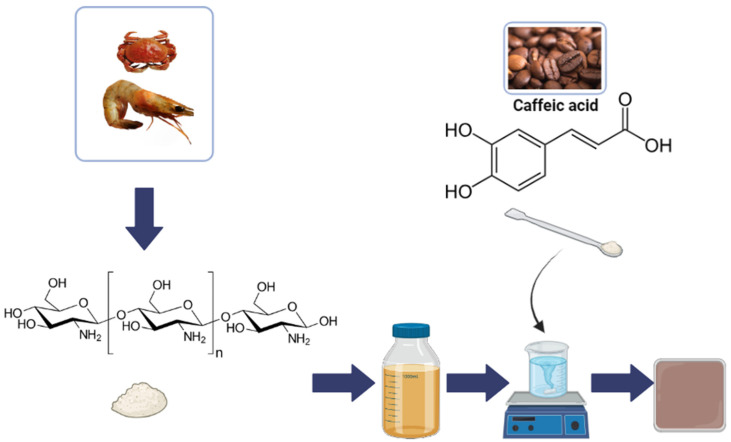
Schematic diagram for the preparation of CS/CA films.

**Figure 2:**
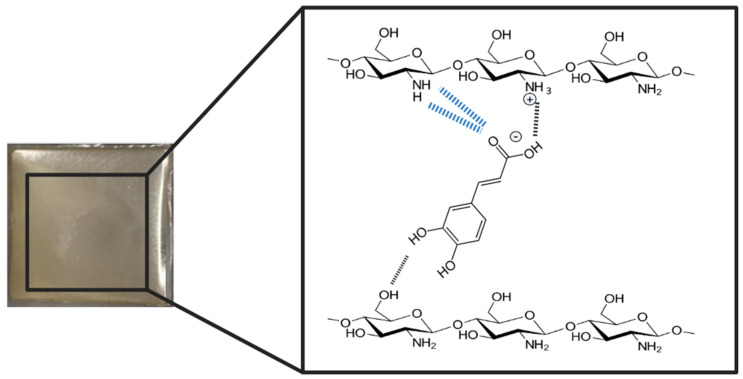
The schematic cross-linking process between CS and CA.

For each type of film, including CS and CS/CA, the neutralization process was carried out by immersing the film in a 1% *w*/*w* NaOH solution for 15 min. The films were then placed in distilled water, which was changed several times until a pH of 7 was reached, and the films were left overnight. In the last step, the films were dried again. The process is illustrated in Figure 1 and Figure 2.

### 2.3. Infrared Spectroscopy

The infrared spectra of CS and CS/CA films before and after alkaline treatment were recorded using a spectrophotometer (Nicolet iS10, Thermo Scientific, Waltham, MA, USA) in attenuated total reflectance (ATR) mode with a diamond as the crystal. The spectral range was 4000–400 cm^−1^. One hundred scans were taken with a spectral resolution of 2 cm^−1^. The infrared spectra of all the films were analyzed using Omnic 9.3.30 software (Thermo Scientific, USA).

### 2.4. Scanning Electron Microscope

A scanning electron microscope (SEM, Quanta 3D FEG, D9399, FEI, Eindhoven, The Netherlands) was used to analyze the surface morphology of the CS and CS/CA films before and after alkaline treatment. The samples were coated with silver to provide a conductive surface for electron beam interaction.

### 2.5. Atomic Force Microscopy

An atomic force microscope (Nanoscope IIIa Multimode Scanning Probe Microscope, Digital Instruments, Veeco Metrology Group, Santa Barbara, CA, USA) was used to analyze the surface images, operating in tapping mode at room temperature in an air atmosphere. The roughness parameter examined as the root-mean-square (Rq) was calculated for the scanned area (5 μm × 5 μm) using NanoScope Analysis v1.40 software (Bruker, Ettlingen, Germany).

### 2.6. Thermogravimetric Analysis

The thermal stability and degradation profiles of the films were measured using an SDT 2960 Simultaneous TGA-DTA analyzer from TA Instruments over a temperature range from 20 °C to 600 °C at a heating rate of 20 °C/min under a nitrogen atmosphere.

### 2.7. Mechanical Properties

The Young’s modulus (YM), and elongation at break (EB) were measured under room conditions using a Z.05 materials testing machine (Zwick Roell, Ulm, Germany). The size of the sample was 10 mm in width and 25 mm in parallel length. A total of five samples of each kind of film were tested.

### 2.8. Swelling Behavior

The swelling behavior of CS films was evaluated at 37 °C in phosphate buffered saline (PBS). The films, with dimensions of 1 cm by 1 cm, were first dried in a vacuum oven at 45 °C for 48 h. Subsequently, they were immersed in PBS at a pH of 7.4 and a temperature of 37 °C [26,27]. After incubation for specified time intervals (1, 4, 24, and 48 h), the films were gently dried by placing them between two sheets of paper and weighed. The swelling ratio (Q) of the films was calculated using Equation (1):

(1) $$Q=\frac{m_{\;}{}_{w}-m_{d}}{m_{d}}*100\%,\;$$

where m_w_ is the weight of the wet film and m_d_ is the weight of the dry film. Three samples of each film type were tested, and the mean values were taken as the swelling ratio.

### 2.9. Contact Angle Measurement

The contact angles of two liquids (glycerin and diiodomethane) on the films were measured using a goniometer equipped with a drop shape analysis system (DSA 10 Control Unit, Krüss, Germany). The contact angle measurements were utilized to calculate the surface free energy (IFT) and its polar (IFT (s,P)) and dispersive (IFT (s,D)) components using the Owens–Wendt method [28].

## 3. Results

### 3.1. Infrared Spectroscopy

**Figure 3:**
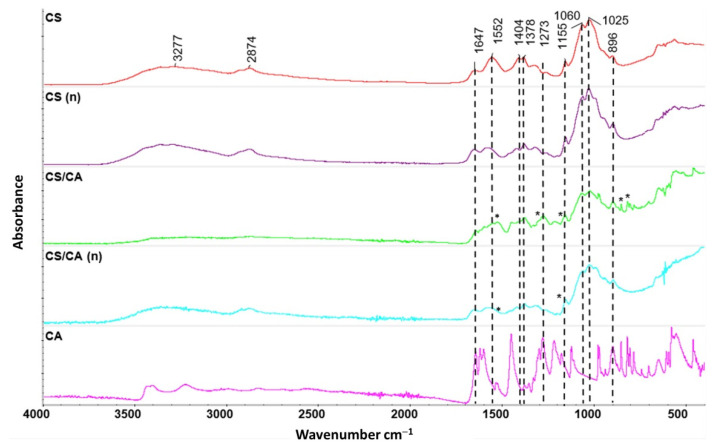
ATR-FTIR spectra of CS and CS/CA films before and after (n) alkaline treatment; asterisks (*) indicate changes in the characteristic bands.

The chemical structural changes in the CS and CS/CA films before and after NaOH treatment were verified using FTIR spectroscopy in ATR mode; the corresponding infrared spectra are depicted in Figure 3. In the CS film, the following bands were observed: the O-H and N-H stretching vibrations at 3277 cm^−1^, the C-H stretching vibrations at 2874 cm^−1^, the amine I band at 1647 cm^−1^, the amine II band at 1552 cm^−1^, the—NH_2_ group vibrations at 1404 cm^−1^, the amine III band at 1378 cm^−1^, the β-1,4-glycosidic bond at 1115 cm^−1^, and the C-O-C stretching vibrations at 1022 cm^−1^.

After the addition of CA, significant differences were observed in the shape and intensity of peaks between 1200 and 1650 cm^−1^, as well as in the bands in the region of 800–900 cm^−1^. These observed bands can be attributed to the presence of the carboxyl group and the aromatic ring of CA. Specifically, the vibrations of the C=O group were observed at 1641 cm^−1^, the stretching vibrations of C=C bonds in the range of 1450–1600 cm^−1^, the stretching vibrations of C-O and C-C bonds in the range of 1200–1300 cm^−1^, and the vibrations of C-H bonds in the range of 800–900 cm^−1^ [21,29,30,31,32]. Additionally, small new peaks were observed for CS/CA films (Figure 3, marked with an asterisk), suggesting that there was an interaction between CS and CA, likely occurring through the formation of hydrogen bonds and electrostatic interactions (as depicted in Figure 2).

### 3.2. SEM

**Figure 4:**
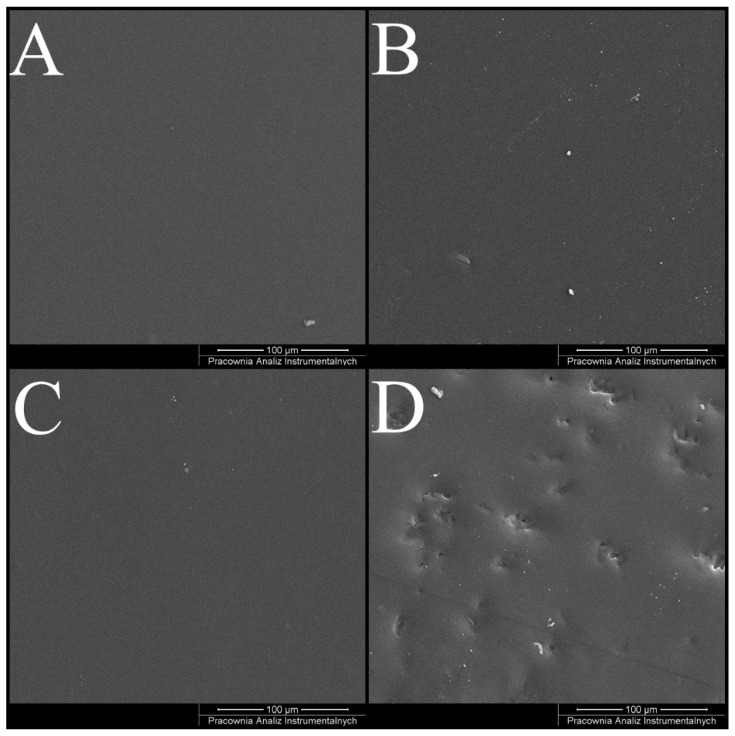
Representative SEM images for film of various compositions: (**A**) CS, (**C**) CS/CA before and (**B**) CS, (**D**) CS/CA after NaOH treatment.

Figure 4 illustrates the SEM images of the CS and CS/CA films before and after NaOH treatment. In the case of CS films, the surface morphology remained relatively consistent before and after neutralization, displaying a homogeneous and comparatively smooth appearance. However, for the CS/CA films, it became evident that domain formation occurred following the NaOH treatment. This phenomenon could be attributed to the leaching of residual solvents and the release of unbound CA constituents.

### 3.3. Atomic Force Microscopy

**Figure 5:**
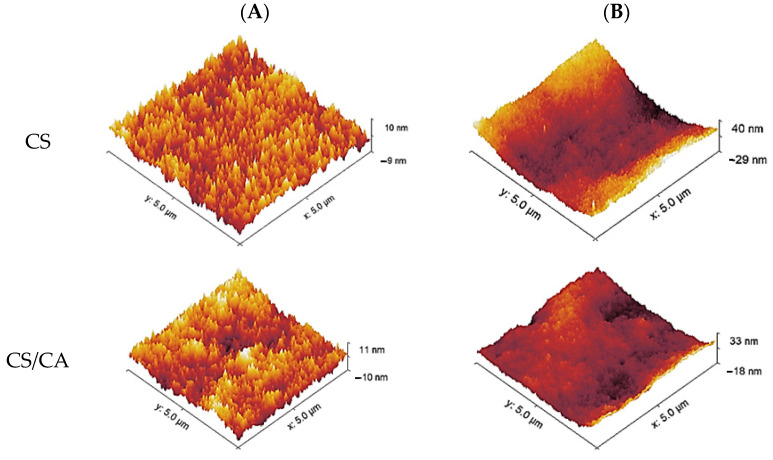
Representative AFM images for film surfaces (**A**) before and (**B**) after NaOH treatment.

Figure 5 presents the atomic force microscopy (AFM) images of the CS and CS/CA films before and after the alkaline treatment. The AFM images revealed a noticeable increase in the quantity and size of domains on the films’ surface following the removal of the acid through NaOH treatment. The films’ surface underwent a transformation toward higher roughness after neutralization, resulting in a corresponding increase in the roughness parameters, as indicated in Table 1. In contrast, prior to NaOH treatment, the roughness parameters of CS and CS/CA films were similar. However, after neutralization, the roughness parameter of the CS/CA film was notably lower than that of CS.

### 3.4. Thermogravimetric Analysis

**Figure 6:**
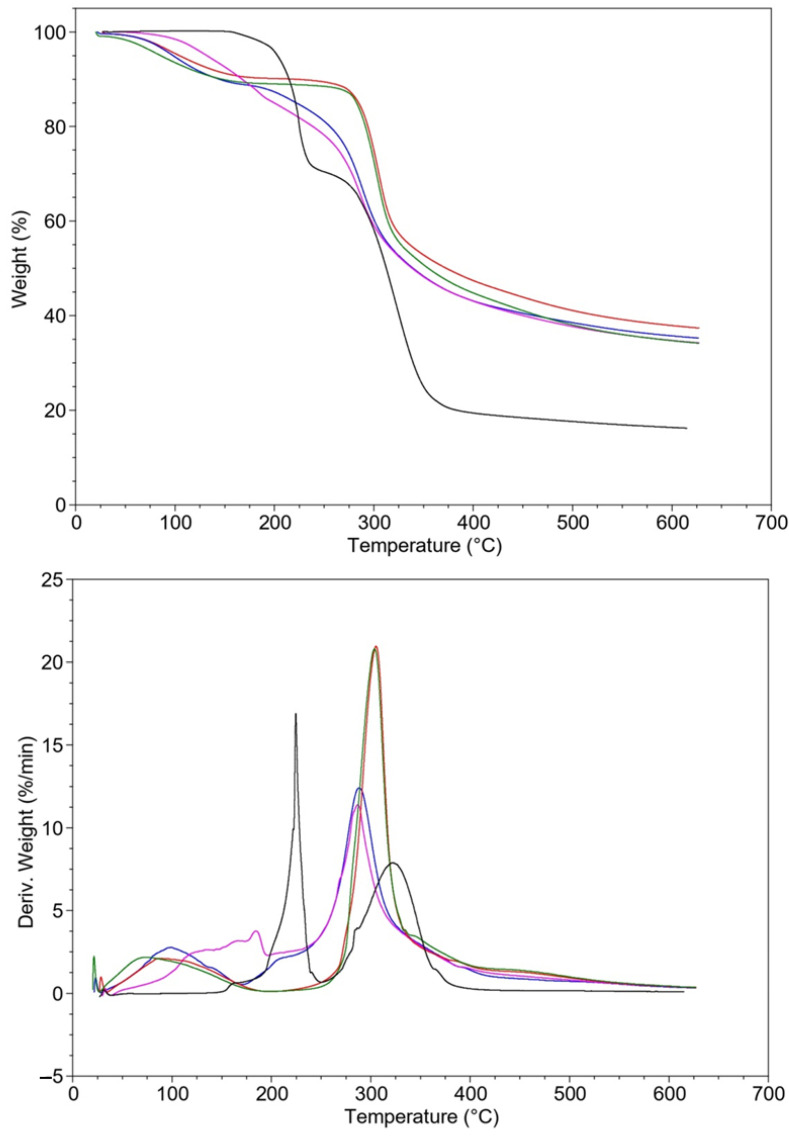
TGA thermograms of CS (blue), CS/CA (purple) films before, and CS (red), CS/CA (green) after NaOH treatment and CA powder (black).

Thermogravimetric analysis (TGA) was conducted to assess the thermal stability of the CS and CS/CA films before and after alkaline treatment, and the corresponding TGA curves are presented in Figure 6. The TGA thermograms of both CS and CS/CA films exhibited two distinct degradation stages. The initial stage involved the elimination of water and residual acid, including acetic acid, up to approximately 250 °C, while the secondary stage occurred between 250 °C and 400 °C. The TGA analysis of CA powder showed degradation occurring over two temperature intervals: 150–250 °C and 250–400 °C. The first stage of weight loss involved both the melting and degradation of CA, while the second decomposition step at higher temperatures may be attributed to the decarboxylation of the acid [33,34]. This indicated that the thermal behavior of CA, both in powder form and when incorporated into the films, was complex and involved multiple degradation processes.

Analyzing the thermograms of the films after neutralization, the first stage, up to 200 °C, indicated the removal of residual acids. In the subsequent phase of maximum weight loss, no significant shift towards higher temperatures was observed for both the pre- and post-neutralization films. This observation suggests that the cross-linking interaction with CA was neither permanent nor stable.

### 3.5. Mechanical Properties

**Figure 7:**
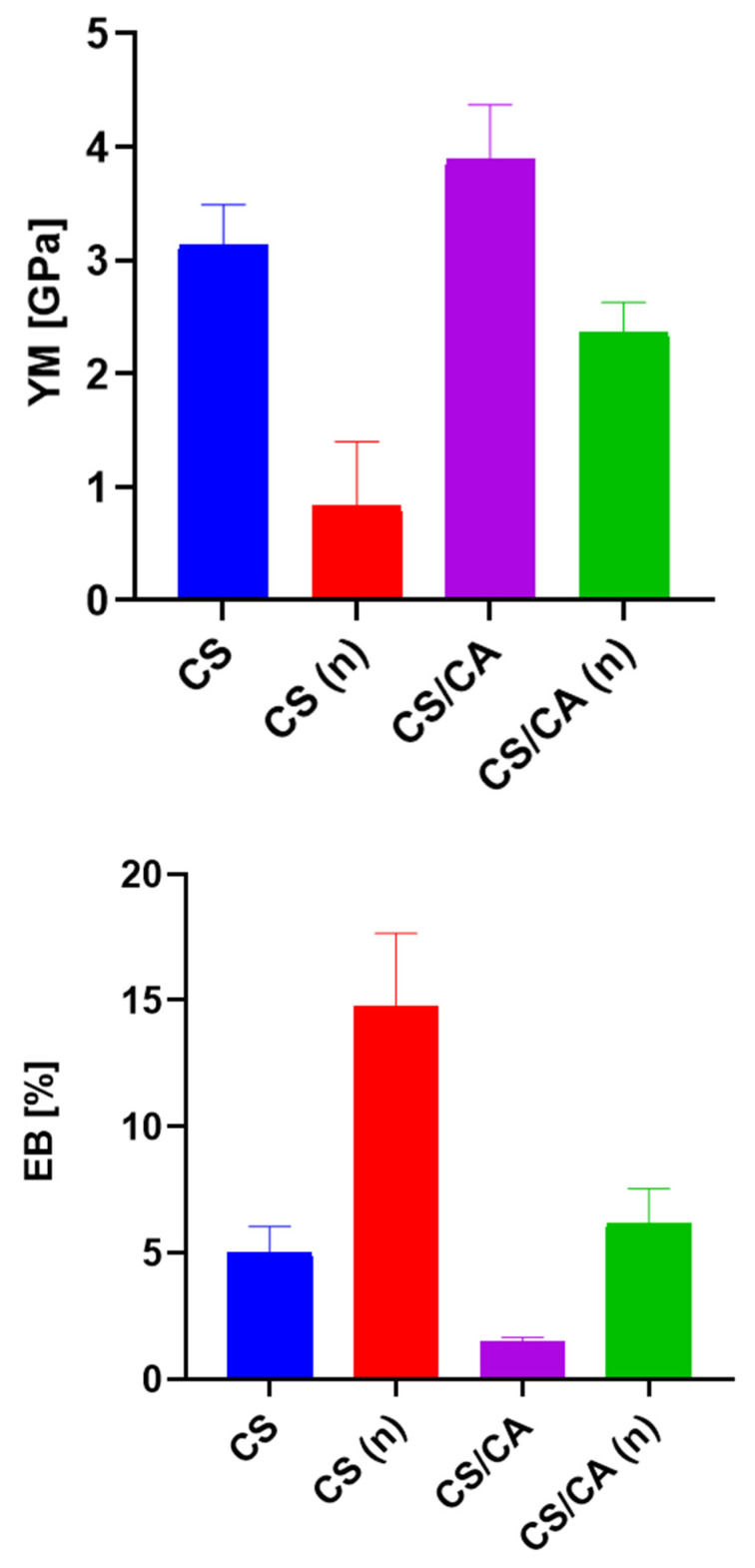
The mechanical parameters (YM and EB) of CS and CS/CA films before and after (n) NaOH treatment, *n* = 5, mean ± SD (standard deviation), error bars represent SD.

Figure 7 shows the relationship between the mechanical parameters, such as YM and EB, and the film compositions. After neutralization, the films experienced a decrease in YM and an increase in EB compared to the films without alkaline treatment. However, in the case of the CS/CA complex, the addition of a cross-linking agent resulted in an increase in Young’s modulus and a decrease in elongation at break. This observation could be attributed to the formation of hydrogen bonds between CS and CA, which enhanced the structural integrity of the film and led to improved mechanical properties.

### 3.6. Swelling Behavior

**Figure 8:**
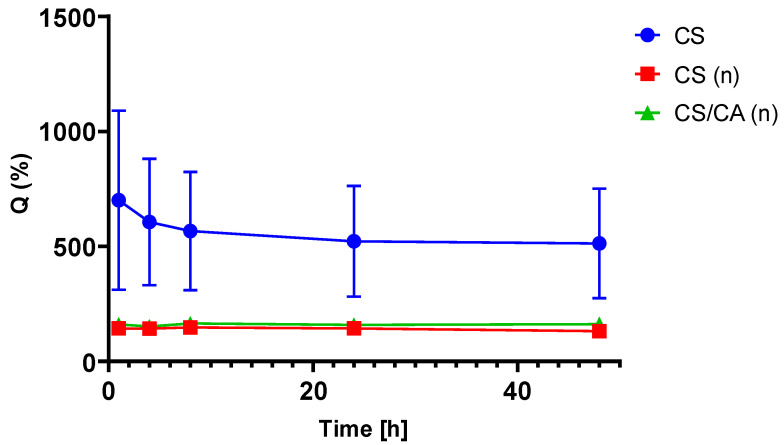
Swelling ratio of CS and CS/CA films before and after (n) NaOH treatment, *n* = 5, mean ± SD (standard deviation).

The swelling behavior of the CS and CS/CA films before and after the neutralization process was investigated by measuring the film weights before and after immersion in a PBS solution (pH = 7.4) at 37 °C. The swelling plots of the films are presented in Figure 8.

**Figure 9:**
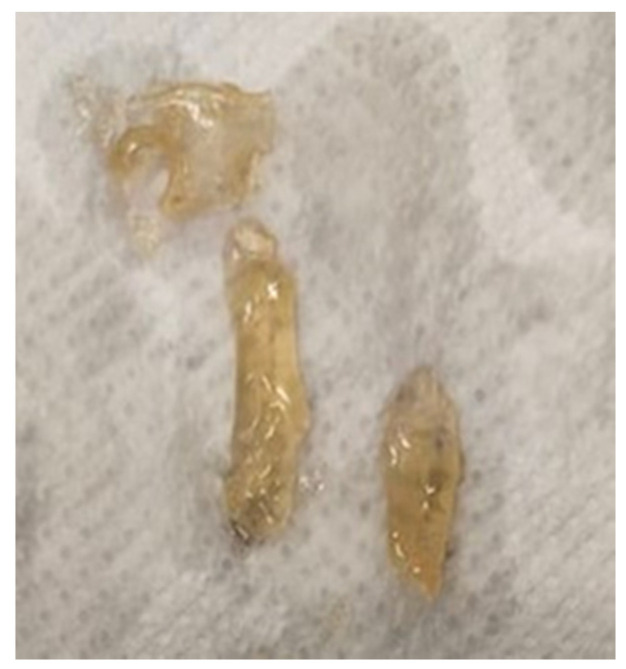
CS/CA hydrogel after 1 h in PBS solution.

The CS films demonstrated the most pronounced swelling rate (Figure 8) throughout each observed period. In the case of CS/CA, the formation of a hydrogel was discerned after one hour of observation (Figure 9). Unfortunately, it could not be weighed. This phenomenon is most likely correlated with the excessive liberation of the free acids, i.e., CA and acetic acid. Following neutralization, the films exhibited a consistent and stable swelling rate over time.

### 3.7. Contact Angle Measurement

The contact angle of two different liquids, glycerin (G) and diiodomethane (D), on the film surface, was studied before and after NaOH treatment. Table 2 presents the corresponding contact angle (θ), surface free energy (IFT (s)), and its polar (IFT (s,P)) and dispersive (IFT (s,D)) components. Analysis of the wetting angle using glycerin and diiodomethane indicated that the incorporation of CA into CS increased the wetting angle for glycerin (Table 2), suggesting that the samples become more hydrophobic. Conversely, the neutralization process made the CS and CS/CA films more hydrophilic in nature. Among all the systems considered, the dispersive component of surface energy prevailed.

## 4. Discussion

CA, a naturally occurring compound, has a significant influence on CS, a biopolymer derived from chitin, when incorporated into its structure. The influence of CA on CS stems from its role as a cross-linking agent, leading to structural modifications within the CS matrix through the formation of hydrogen bonds between the carbonyl and hydroxyl groups of CA and the amino or hydroxyl groups present in the CS molecule. Additionally, electrostatic interactions occur between the charged amino group (NH_3_^+^) of CS and the charged carboxyl group (COO^−^) of CA [22,34].

Polymer films, widely utilized in various applications, can improve their properties through a neutralization process [35]. This process is specifically designed to eliminate any residual solvents and unreacted cross-linking agents, resulting in overall enhancements in film performance. Cross-linking, as a general technique, is employed to enhance the physicochemical properties of polymers by creating additional interconnections within their structures.

When CA is added in combination with the neutralization process, notable transformations occurred in the surface properties of the resulting film. These changes included an increase in surface roughness, which holds significant implications for various applications. These modifications are particularly relevant in the fields of biomedicine and cosmetics, where the material’s full potential benefits can be realized.

The improved elasticity and increased surface roughness of the film make it a promising candidate for applications in soft tissue engineering. In such applications, greater elasticity is highly desirable as it enhances compatibility with the surrounding biological environment. Additionally, the heightened surface roughness of the film promotes enhanced cell adhesion, which is crucial for successful tissue regeneration and integration.

The neutralization process employed in this study showed a weak binding of CA by CS, as evidenced by the results obtained from infrared studies, thermal analysis, and swelling studies. Nevertheless, notable modifications were observed on the films’ surfaces, resulting in increased roughness and alterations in mechanical parameters.

The enhanced hydrophilicity of the films and the increased stability of the swelling factor provide additional advantages for potential cellular applications. However, it is important to note that while the study demonstrated the observed changes in the CS structure and the potential for hydrogen bonding and electrostatic interactions, the results also indicated the need for further modification of these materials. Additional research is required to achieve a stronger cross-linking effect between CS films and CA using other conditions for the neutralization process and/or the addition of other compounds.

## 5. Conclusions

In this study, CS films were prepared and characterized with and without the addition of CA, both before and after the neutralization process. The research findings regarding morphology, structural, mechanical, thermal, and surface properties clearly demonstrated intermolecular interactions between CS and CA. The incorporation of CA into CS films resulted in improved elasticity, while alkaline treatment induced significant surface changes, thus leading to increased roughness when compared to the initially smooth surface of CS films and the largest increase being observed in pure CS films. Furthermore, all the CS films neutralized with NaOH exhibited higher stiffness when compared to their pre-neutralization state.

In addition, this study revealed a weak interaction between CA and CS, which led to the removal of CA from the films during the neutralization process, as shown by the studies on film swelling behavior. Additionally, the removal of acetic acid and CA from the films resulted in a different structure compared to the case of acetic acid alone, which impacted the studied properties of the films. These results indicate the potential for further modification of these materials, contributing to the development of advanced biomaterials. Other approaches to enhance the properties of CS materials with CA could involve using a different method to add CA to the polymer matrix, such as utilizing a CA solution, altering the temperature during film formation, and employing milder neutralization conditions. These topics will be investigated in our future research. The addition of CA to CS films may enhance their adhesion to the skin, making them valuable in various biomaterial applications. Moreover, CA possesses antioxidant and anti-inflammatory properties, further enhancing the potential of CS films incorporating this compound.

## Figures and Tables

**Table 1:** Roughness parameters of CS and CS/CA films before and after NaOH treatment.

Sample	Before	After
	R_q_ (nm)	R_q_ (nm)
CS	2.49	11.92
CS/CA	2.96	6.42

**Table 2:** The contact angle for glycerin (θ^G^), for diiodomethane (θ^D^), surface free energy—IFT (s), its polar—IFT (s,P) and dispersive—IFT (s,D) components of CS and CS/CA films before and after (n) neutralization, *n* = 5, mean ± SD (standard deviation).

Sample	θG [°]	θD [°]	IFT (s) [mJ/m^2^]	IFT (s,D) [mJ/m^2^]	IFP(s,P) [mJ/m^2^]
CS	55.45 ± 1.45	61.64 ± 5.73	37.23 ± 3.06	23.21 ± 0.28	14.03 ± 3.34
CS (n)	41.50 ± 1.49	64.70 ± 2.24	40.56 ± 1.19	32.19 ± 0.31	8.37 ± 0.89
CS/CA	60.00 ± 0.88	58.10 ± 5.75	38.00 ± 3.35	19.82 ± 0.52	18.18 ± 3.87
CS/CA (n)	42.32 ± 1.76	65.38 ± 3.59	40.08 ± 1.67	31.88 ± 0.14	8.20 ± 1.53

## Data Availability

Data are contained within the article.
